# Internetwork Connectivity Predicts Cognitive Decline in Parkinson’s and Is Altered by Genetic Variants

**DOI:** 10.3389/fnagi.2022.853029

**Published:** 2022-03-28

**Authors:** Xiangyu Wei, Qian Shen, Irene Litvan, Mingxiong Huang, Roland R. Lee, Deborah L. Harrington

**Affiliations:** ^1^Research and Radiology Services, VA San Diego Healthcare System, San Diego, CA, United States; ^2^Revelle College, University of California San Diego, La Jolla, CA, United States; ^3^Department of Radiology, University of California San Diego, La Jolla, CA, United States; ^4^Department of Neurosciences, University of California San Diego, La Jolla, CA, United States

**Keywords:** Parkinson’s disease, internetwork functional connectivity, cognition, MAPT, SNCA, genetic variants, resting state fMRI, independent component analyses

## Abstract

In Parkinson’s disease (PD) functional changes in the brain occur years before significant cognitive symptoms manifest yet core large-scale networks that maintain cognition and predict future cognitive decline are poorly understood. The present study investigated internetwork functional connectivity of visual (VN), anterior and posterior default mode (aDMN, pDMN), left/right frontoparietal (LFPN, RFPN), and salience (SN) networks in 63 cognitively normal PD (PDCN) and 43 healthy controls who underwent resting-state functional MRI. The functional relevance of internetwork coupling topologies was tested by their correlations with baseline cognitive performance in each group and with 2-year cognitive changes in a PDCN subsample. To disentangle heterogeneity in neurocognitive functioning, we also studied whether α-synuclein (SNCA) and microtubule-associated protein tau (MAPT) variants alter internetwork connectivity and/or accelerate cognitive decline. We found that internetwork connectivity was largely preserved in PDCN, except for reduced pDMN-RFPN/LFPN couplings, which correlated with poorer baseline global cognition. Preserved internetwork couplings also correlated with domain-specific cognition but differently for the two groups. In PDCN, stronger positive internetwork coupling topologies correlated with better cognition at baseline, suggesting a compensatory mechanism arising from less effective deployment of networks that supported cognition in healthy controls. However, stronger positive internetwork coupling topologies typically predicted greater longitudinal decline in most cognitive domains, suggesting that they were surrogate markers of neuronal vulnerability. In this regard, stronger aDMN-SN, LFPN-SN, and/or LFPN-VN connectivity predicted longitudinal decline in attention, working memory, executive functioning, and visual cognition, which is a risk factor for dementia. Coupling strengths of some internetwork topologies were altered by genetic variants. PDCN carriers of the SNCA risk allele showed amplified anticorrelations between the SN and the VN/pDMN, which supported cognition in healthy controls, but strengthened pDMN-RFPN connectivity, which maintained visual memory longitudinally. PDCN carriers of the MAPT risk allele showed greater longitudinal decline in working memory and increased VN-LFPN connectivity, which in turn predicted greater decline in visuospatial processing. Collectively, the results suggest that cognition is maintained by functional reconfiguration of large-scale internetwork communications, which are partly altered by genetic risk factors and predict future domain-specific cognitive progression.

## Introduction

Cognitive decline is common in early stages of Parkinson’s disease (PD), but diversity exists in the domains affected suggesting that patterns of neurodegeneration differ amongst people. The pathophysiological underpinnings of cognitive changes are complex, involving multiple neurotransmitter systems (Gratwicke et al., 2015) and large-scale brain networks (Tessitore et al., 2019), which may be vulnerable to genetic variants that carry different risks for neurocognitive progression including α-synuclein (SNCA) (Sampedro et al., 2018a; Ramezani et al., 2021) and microtubule-associated protein tau (MAPT) (Williams-Gray et al., 2009; Morley et al., 2012; Sampedro et al., 2018b). Knowledge about the pathophysiology behind cognitive changes in PD is largely based on people who already show mild cognitive impairment (MCI). Yet changes in the brain occur years before cognitive symptoms manifest (Harrington et al., 2020, 2021). As optimal treatments depend on early detection, markers of brain dysfunction that predate MCI and foreshadow the evolution of cognitive decline are sorely needed.

Across neurological disorders, intrinsic functional connectivity during resting-state fMRI (rsfMRI) is altered within core large-scale functional networks that can be identified using independent component analyses (ICA), a data-driven technique. The default mode network (DMN) is associated with internally generated cognitive states including memory retrieval, and was called a task-negative network as activity is often suppressed during task-evoked fMRI (Di and Biswal, 2014). Task-positive intrinsic networks include the salience network (SN), which controls attentional capture, internetwork switching, and inhibition (Uddin, 2015), and the left and right frontoparietal networks (LFPN, RFPN), which govern working memory and executive control (Grady et al., 2016). Although intrinsic connectivity disturbances within these and other large-scale networks have been studied in cognitively normal PD (PDCN) (Tessitore et al., 2012; Amboni et al., 2015; Baggio et al., 2015; Gorges et al., 2015; Peraza et al., 2017; Hou et al., 2021), findings are mixed likely due to diverse criteria for cognitive impairment and small samples of some studies.

Less attention has been paid to large-scale internetwork communications in PDCN, despite their role in integrating and segregating different processing resources to support cognition (Bressler and Menon, 2010). In a non-demented PD cohort, SN-RFPN and DMN-RFPN couplings were, respectively, reduced and increased relative to controls (Putcha et al., 2015). Visual network (VN) internetwork couplings were also reduced in a mixed PDCN/MCI cohort (Hou et al., 2021), in alignment with visual cognition disturbances in PD (Weil et al., 2016). However, little is known about potential large-scale internetwork disturbances in PDCN. Seed-based approaches, which measure the strength of connectivity between regions of interest, suggest that FPN regional couplings with DMN, dorsal attention (DAN), and/or VN regions are altered in PDCN (Baggio et al., 2015; Klobusiakova et al., 2019), yet normal in a mixed PDCN/MCI cohort (Campbell et al., 2020). The strength of FPN-DMN and FPN-VN regional couplings also increased over 1 year in PDCN and PD-MCI cohorts (Klobusiakova et al., 2019). Altogether, it appears that aberrant FPN internetwork communications may be a prominent feature of PD, despite discrepancies in interacting networks, which could be linked to different analytic techniques (Koch et al., 2010; Badea et al., 2017). The cognitive relevance of internetwork couplings, however, has received scant attention in PDCN.

The present study investigated internetwork intrinsic functional connectivity in PDCN and healthy adults in core networks including the DMN, LFPN, RFPN, and SN. As the DMN is composed of subsystems that capture the heterogeneous nature of self-generated thoughts (Andrews-Hanna et al., 2014b), we studied the anterior DMN (aDMN), which supports self-generated executive, conceptual, and semantic processes relevant to PD (Harrington et al., 2020, 2021), and the posterior DMN (pDMN) which supports episodic and autobiographical memory, and associative or constructive processes. VN internetwork connectivity was studied as well since disturbances in visual cognition are a marker of future dementia (Williams-Gray et al., 2013). The functional relevance of internetwork coupling topologies was tested by their correlations with baseline cognitive functioning. We also examined whether internetwork coupling topologies predicted 2-year changes in cognition in a PDCN subsample. To unravel heterogeneity in neurocognitive functioning, we examined whether genetic variants modulated internetwork connectivity and accelerated cognitive decline. We predicted that SNCA expression would alter SN internetwork connectivity owing to accumulation of α-synuclein in the insula (Christopher et al., 2014). Tau expression was expected to alter internetwork topologies comprised of frontal and posterior cortices (FPN, DMN, VN), which are modulated by MAPT variants (Nombela et al., 2014; Harrington et al., 2021).

## Materials and Methods

### Participants

Participants were 63 PDCN who met the PD United Kingdom Brain Bank Criteria and 43 age, sex and education matched healthy controls. Exclusion criteria included neurological diagnoses other than PD, psychiatric diagnoses, history of alcohol or substance abuse, metal in the head, positive MRI findings, use of anticholinergics or cognitive medications, and cognitive complaints. PD volunteers with tremors or dyskinesias that might cause head motion were excluded. Volunteers were excluded if they met the Movement Disorders Society Level II criteria for PD-MCI (Litvan et al., 2012). MCI was defined as > 1.5 standard deviations below the control group mean on at least two tests in single or different domains and the absence of cognitive complaints. There were six *de novo* PD, five taking dopamine agonist monotherapy, 26 taking levodopa monotherapy, and 26 taking levodopa combination therapy. Testing was conducted while on medication to minimize motion artifact during scanning. The Institutional Review Board at the VA San Diego Healthcare System approved the study. All subjects signed written informed consent.

### Neuropsychological Assessments

Participants were administered tests of premorbid intelligence (Wechsler Test of Adult Reading) and the Montreal Cognitive Assessment (MoCA), a dementia screening tool. Table 1 summaries the comprehensive test battery used to screen for MCI (Level II criteria), which contained two tests for each of five domains including 1) attention and working memory (Color-Word Naming, CWN; Adaptive Digit Ordering, DOT), executive functioning (Category Switching, SWA; Color-Word Inhibition, CWINH), visual and verbal memory (California Verbal Learning Test, CVLT; Brief Visuospatial Memory Test, BVMT), visual cognition, which measured visuospatial processing (Judgment of Line Orientation, JLOT) and visual organization (Hooper Visual Organization Test, HVOT), and semantic language, which measured confrontation naming (Boston Naming Test, BNT) and semantic fluency (Category Fluency, CAT). Approximately 2-years post-baseline testing, the same test battery was administered to a subsample of 40 PDCN, but not control participants. For this follow-up visit, alternative forms of the tests were used.

**TABLE 1:** Demographic, clinical, and cognitive characteristics.

	PD (*n* = 63)	HC (*n* = 43)	*p*	η*_*p*_*^2^
Age (years)	65.4 (6.4)	64.1 (8.5)	0.37	0.01
Education (years)	17.0 (2.1)	17.0 (2.1)	0.88	0.00
Sex (% females)	41.30%	44.20%	0.77	
Handedness (% right-handed)	82.50%	88.40%	0.58	
Wechsler test of adult reading	44.5 (5.0)	45.6 (3.8)	0.23	0.01
Montreal Cognitive Assessment (MoCA)	26.9 (2.3)	27.6 (2.0)	0.08	0.03
Disease duration (years)	4.6 (3.8)			
Levodopa dosage equivalence^†^	908.1 (654.0)			
UPDRS Part III	23.1 (11.3)			
**Head motion**				
Maximum rotation (degrees)	0.30 (016)	0.29 (0.19)	0.82	0.00
Maximum translation (mm)	0.58 (0.16)	0.55 (0.16)	0.31	0.01
Mean rotation (degrees)	0.10 (0.06)	0.09 (0.05)	0.25	0.01
Mean translation (mm)	0.22 (0.06)	0.21 (0.06)	0.21	0.02
**Genetic variants**				
SNCArs356219 AA:AG:GG	14:34:15	15:24:4	0.11	
MAPTrs242557 GG:GA:AA	30:25:8	13:22:8	0.20	
**Attention and working memory**				
Adaptive Digit Ordering (DOT)	6.5 (1.9)	6.6 (2.2)	0.70	0.00
DKEFS Color + Word Naming (CWN)	21.5 (4.4)	21.8 (4.5)	0.70	0.00
**Executive functioning (DKEFS)**				
Category switching (accuracy) (SWA)	13.5 (2.8)	13.3 (3.1)	0.72	0.00
Color-Word Inhibition (CWINH)	59.2 (12.9)	56.2 (11.3)	0.22	0.01
**Memory (long delay free recall)**				
California Verbal Learning Test 2 (CVLT)	9.0 (3.4)	11.3 (3.0)	0.001	0.11
Brief Visuospatial Memory Test-Revised (BVMT)	8.3 (2.5)	9.9 (1.9)	0.001	0.11
**Visual cognition**				
Judgment of Line Orientation (JLOT)	25.4 (2.9)	26.9 (2.7)	0.008	0.07
Hooper Visual Organization (HVOT)	25.4 (2.3)	26.9 (1.7)	0.001	0.10
**Semantic language**				
Boston Naming (BNT)	57.6 (2.6)	58.3 (1.7)	0.12	0.02
DKEFS Category Fluency (CAT)	43.2 (8.4)	44.2 (9.1)	0.55	0.00

*Tabled values are raw score means (standard deviations). Group differences were tested using ANOVA and Pearson chi-square statistics (sex, handedness, SNCA, MAPT). UPDRS, Movement Disorder Society Unified Parkinson’s Disease Rating Scale; DKEFS, Delis Kaplan Executive Function System.*

*^†^Levodopa dosage equivalence was calculated using the method of Tomlinson (Tomlinson et al., 2010). Data are based on 57 participants who were taking dopaminergic therapy.*

Table 1 shows that the groups did not differ in demographics, premorbid intelligence, or MoCA scores. Although some participants in both groups had scores that were lower than the traditional cutoff score of 26 for probable MCI, this cutoff leads to a high percentage of false positives (Weintraub et al., 2015; Carson et al., 2018; Faust-Socher et al., 2019). Indeed, many people with lower MoCA scores (22–25) show normal neuropsychological testing, and people with high MoCA scores (26–30) can show MCI on neuropsychological testing (Rosenblum et al., 2020). That said, no participant with lower MoCA scores exhibited MCI using comprehensive neuropsychological testing, which is the gold standard. The PDCN group had significantly lower scores than controls on verbal and visual memory long delay free recall (CVLT; BVMT) and both tests of visual cognition (JLOT; HVOT). This indicates a decline at the group level, but individual participants did not exhibit cognitive decline indicative of MCI.

### Genotyping

Oragene-500 kits^1^ were used to collect whole saliva samples (2 mL). TaqMan assays were used to genotype SNCA and MAPT polymorphisms relevant to PD (Compta et al., 2011; Nalls et al., 2011; Zhang et al., 2018). MAPTrs242557 codes the intra-H1 variation in transcriptional activity. The A allele associated with higher tau transcription levels than the G allele (Compta et al., 2011; Chen et al., 2017). SNCA rs356219 codes variations in α-synuclein levels. The G allele is linked to higher transformed plasma α-synuclein levels (Emelyanov et al., 2018).

### Imaging Protocols

Imaging was conducted on a GE MR750 Discovery 3 Tesla system equipped with a Nova Medical 32-channel head coil. Head motion was limited by foam pads inserted between the head and the coil. High-resolution T1-weighted anatomical images maximized differentiation of the white and gray matter boundary (3D spoiled gradient-recalled at steady state, minimum full TE, 3.5 ms; TR, 2,852 ms; TI, 1,000 ms; 8° flip angle; 0.8-mm slices, acquisition matrix = 512). The rsfMRI images used a high spatial and temporal resolution multiband accelerated gradient EPI sequence (slice thickness = 2 mm; TR = 800 ms; TE = 35 ms; flip angle = 52°; acquisition matrix = 104; axial slices = 72; multiband factor = 8; echo spacing = 0.612 ms; band width = 4807.69 Hz/Px), which has greater sensitivity and specificity than conventional single-band EPI protocols (Tomasi et al., 2016). Three rsfMRI runs were acquired, each lasting 5 min and 35 s. The first 12.8 s were removed to allow magnetization to stabilize to a steady state. To correct geometric distortions, a pair of gradient EPI sequences with anterior and posterior reversed gradients (TR = 8,500 ms; TE = 70.6 ms; isotropic voxels = 2 mm; flip angle = 90°; echo spacing = 0.612 ms) were acquired before rsfMRI runs.

### Image Preprocessing

Functional images were preprocessed using FSL 6.4^2^ and Analysis of Functional Imaging 20.0.^3^ First a field map was computed from the pair of anterior and posterior reversed gradient sequences. Then it was applied to rsfMRI data to correct geometric distortions using FSL TOPUP. Each subject’s distortion corrected data from three rsfMRI runs were concatenated. Additional preprocessing included motion correction using FSL MCFLIRT, high pass temporal filtering, spatial smoothing [6 mm full width half maximum (FWHM) filter] and registration to T1-weighted images. Data were transformed to MNI atlas space and carefully checked for normalization accuracy. Table 1 shows that there were no group differences in maximum or mean rotation and translation head movements.

### Independent Components Analysis

Preprocessed images were analyzed with MELODIC using a temporal concatenation spatial ICA approach (Beckmann and Smith, 2004). For each subject, 25 temporally and spatially coherent patterns were extracted, clustered at the group level, and then visually inspected for their correspondence to well-characterized networks of interest. Spatial cross-correlation was also performed to cross-validate extracted ICs with resting-state ICN templates (Smith et al., 2009; Laird et al., 2011). Six components of interest were chosen including the VN, aDMN, pDMN, LFPN, RFPN, and SN.

Dual regression analysis was conducted on the six components. First, each component was used as a mask to extract a subject and component-specific mean time course, which describes the temporal dynamics and synchrony of each component network. The time courses were then fed into a linear model against individual rsfMRI data to obtain subject-specific maps of each component (Beckmann et al., 2009; Nickerson et al., 2017). To validate that each component network was expressed in both the PD and control groups, voxelwise one sample *t*-tests were performed for each group using FSL randomize (5,000 permutations) with familywise error (FWE) correction for multiple comparisons (*p* < 0.05) (Nichols and Holmes, 2002). Brain regions with significant *p*-values in both groups in each component network were combined to form a mask to test for group differences in intra-network connectivity (FSL randomize, 5,000 permutations, *p* < 0.05, FWE corrected).

The main analyses focused on group differences in internetwork connectivity. To compute the strength of internetwork functional connectivity, Pearson correlations were performed between the subject-specific time-courses of all possible network pairs, which were extracted from the dual regression analysis for each network. A Fisher z-transformation was applied to the correlation coefficients to obtain a standardized z score of internetwork connectivity for each subject.

### Brain Structure

To determine if internetwork couplings in the PD group were related to brain atrophy, cortical thickness and volume were analyzed using FreeSurfer 5.3.^4^ Individual subject’s cortical folding patterns were inflated, registered to a standard spherical surface template, and smoothed with 10 mm FWHM Gaussian kernel to improve the signal-to-noise ratio and reduce local variations across subjects (Fjell et al., 2009). Tests of group differences in thickness and volume (age as a nuisance variable) were conducted across the continuous cortical surface using the FreeSurfer QDEC application and the false discovery rate (FDR, *q* < 0.05) adjustment for multiple comparisons. Group differences were also tested for subcortical volumes of interest (bilateral caudate, putamen, and hippocampus), which were adjusted for total intracranial volume.

### Statistical Analyses

In total, 15 internetwork coupling topologies were studied. Group differences in internetwork connectivity were tested using independent *t*-tests with bias-corrected accelerated bootstrapping (1,000 iterations) to reliability estimate connectivity distributions. *P*-values were uncorrected due to the large number of internetwork pairs and hence, the results should be cautiously interpreted. For multiple regression analyses, stepwise models entered sets of internetwork connections as the predictors of cognitive variables and genetic variants. First, we analyzed VN internetwork connections with all other networks (aDMN, pDMN, LFPN, RFPN, SN), as the VN has been poorly studied in PD despite disturbances in visual cognition (Weil et al., 2016), which correlate with occipital thinning (Garcia-Diaz et al., 2018). Second, aDMN internetwork couplings with other networks (pDMN, LFPN, RFPN, SN) were analyzed as this subnetwork regulates internally generated executive and semantic processes, which may interact with processing in other networks to support of cognition. Third, we examined pDMN couplings with core task-positive networks (LFPN, RFPN, SN). Fourth, couplings amongst task-positive networks were studied (LFPN-RFPN, LFPN-SN, RPFN-SN) given their roles in attention, working memory, and cognitive flexibility (Seeley et al., 2007), which are relevant to PD. All cognitive variables were converted to age adjusted residuals owing to associations of some variables with age, but not educational level.

For each group, stepwise multiple regressions were performed to identify internetwork couplings that best predicted cognition on tests of global cognition (MoCA), attention (CWN) and working memory (DOT), executive function (SWA, CWINH), visuospatial cognition (JLOT, HVOT), visual and verbal episodic memory (BVMT, CVLT), and semantic cognition (BNT, CAT). Longitudinal changes in cognition were studied in 40 PDCN participants who were retested 24.5 months post baseline (*SD* = 3.2, minimum = 19.9, maximum = 32.8). Longitudinal changes in age adjusted residuals were calculated using simple discrepancy scores (score at time 1—score at time 2). FDR correction was applied to all uncorrected *p*-values from the analyses of both baseline and longitudinal changes in cognition.

ANOVAs with bias-corrected accelerated bootstrapping tested for the effect of gene variants on (1) baseline cognitive variables for each group and (2) longitudinal changes in cognition in the PDCN subsample. To test for internetwork connectivity predictors of gene expression, stepwise multiple regressions tested for sets of internetwork couplings that predicted SNCA and MAPT expression (FDR adjusted).

## Results

### Group Differences in Brain Morphometry

Group differences in cortical thickness and volume were non-significant. Independent *t*-tests (bias-corrected, bootstrapped) also failed to show group differences in left and right putamen, caudate, and hippocampus volumes (FDR adjusted). Due to the lack of manifested atrophy in the PDCN group relative to controls, gray matter was not used as a covariate in subsequent analyses.

### Group Differences in Intra-Network Connectivity

**FIGURE 1:**
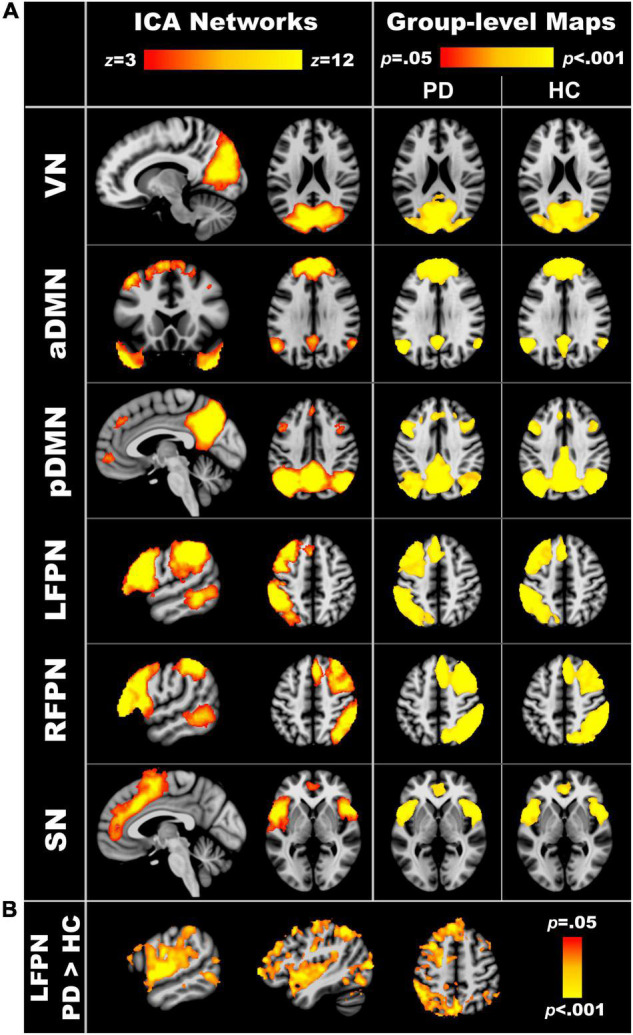
Resting-state networks of interest. **(A)** The left column displays the spatial maps obtained from independent component analyses of the entire sample. The right column shows the group level maps derived from the dual regression analyses. To validate that each of the six component networks were expressed in both the PD and healthy control groups, voxelwise one sample *t*-tests were performed separately for each group using familywise error (FWE) correction for multiple comparisons (*p* < 0.05). **(B)** Significant group differences in intra-network connectivity of the LFPN (*p* < 0.05, FWE corrected). VN, visual network; aDMN, anterior default mode network; pDMN, posterior default mode network; LFPN, left frontoparietal network; RFPN, right frontoparietal network; SN, salience network.

Figure 1A displays the components of interest from the group ICA analyses. Supplementary Table 1 lists the main regions comprising each component. Each of the six components was significantly expressed in the PDCN and control groups (one sample *t*-tests, *p* < 0.05). Figure 1B displays the results from between-group voxelwise comparisons of intra-network connectivity (two sample *t*-tests; *p* < 0.05). Only LFPN intra-network couplings were stronger in PDCN (*z* = 6.8, *SD* = 3.7) than controls (*z* = 4.7, *SD* = 2.8) [*F*(1, 104) = 10.5, *p* < 0.002, η*_*p*_*^2^ = 0.09]. In the PDCN group, aberrant LFPN intra-network coherence (z scores) did not correlate with baseline or longitudinal changes in cognition.

### Group Differences in Internetwork Connectivity

Independent *t*-tests (bootstrapped) tested for group differences in internetwork connectivity. Table 2 shows that internetwork couplings were stronger in the control than the PDCN group for the pDMN-LFPN [*t*_(1, 104)_ = 2.0, *p* = 0.047, *d* = 0.40], pDMN-RFPN [*t*_(1, 104)_ = 2.41, *p* = 0.018, *d* = 0.48], and at a subthreshold level of significance, the LFPN-RFPN [*t*_(1, 104)_ = 1.96, *p* = 0.053, *d* = 0.39]. Nonetheless, the findings should be cautiously interpreted as uncorrected *p*-values did not survive FDR correction. In the PDCN group, LFPN internetwork couplings did not correlate with aberrant LFPN intra-network coherence (z scores).

**TABLE 2:** Group differences in internetwork functional connectivity.

	PDCN (*n* = 63)	Controls (*n* = 43)	*p*	Cohen’s *d*
**Visual network**				
VN-aDMN	−1.9(3.9)	−1.7(3.8)	0.80	0.05
VN-pDMN	2.8 (4.8)	2.0 (3.3)	0.40	0.17
VN-LFPN	−0.5(4.3)	−0.3(3.7)	0.73	0.07
VN-RFPN	2.4 (3.3)	3.3 (3.1)	0.13	0.30
VN-SN	−1.6(3.4)	−1.2(3.5)	0.59	0.11
**Anterior default mode network**				
aDMN-pDMN	2.4 (3.6)	2.7 (3.8)	0.73	0.07
aDMN-LFPN	2.2 (4.1)	1.4 (3.5)	0.26	0.22
aDMN-RFPN	1.2 (3.4)	1.5 (3.6)	0.74	0.07
aDMN-SN	1.3 (3.4)	0.5 (3.6)	0.27	0.22
**Posterior default mode network**				
pDMN-LFPN	2.0 (4.0)	3.5 (3.1)	**0.047**	0.40
pDMN-RFPN	1.0 (2.9)	2.4 (3.0)	**0.018**	0.48
pDMN-SN	−3.1(3.7)	−3.0(3.4)	0.92	0.02
**Frontoparietal network**				
LFPN-RFPN	2.5 (4.5)	4.1 (3.8)	0.053	0.39
LFPN-SN	1.8 (3.2)	0.9 (3.2)	0.14	0.29
RFPN-SN	0.3 (3.4)	0.6 (3.3)	0.67	0.09

*Tabled values are Fisher z score means (standard deviations). Group differences were tested using independent t-tests with bias-corrected accelerated bootstrapping (1,000 iterations). Nominally significant p-values (bold) were not significant after FDR correction.*

*VN, visual network; aDMN, anterior default mode network; pDMN, posterior default mode network; LFPN, left frontoparietal network; RFPN, right frontoparietal network; SN, salience network.*

### Internetwork Connectivity Associations With Baseline Cognition

**FIGURE 2:**
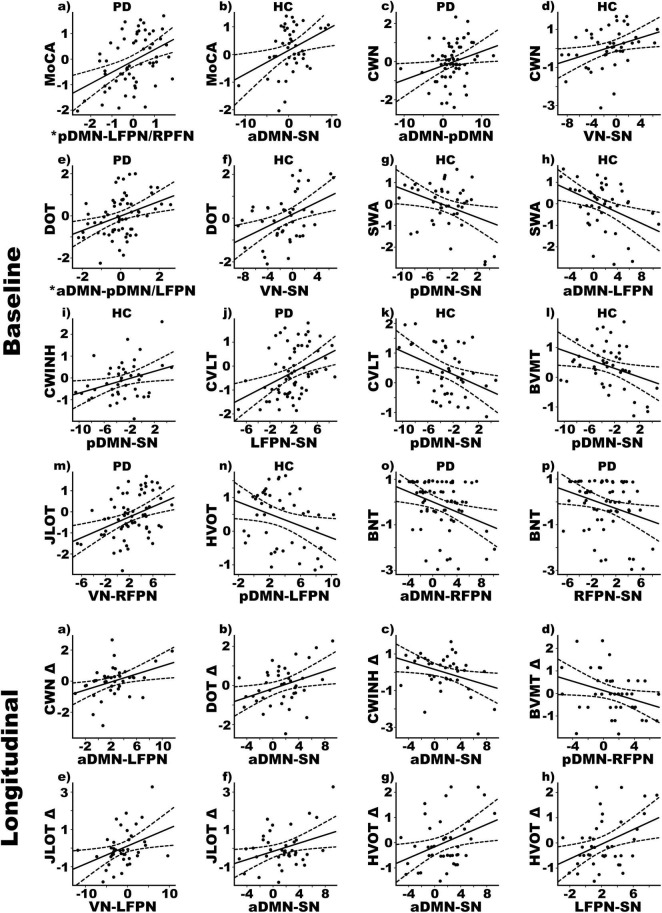
Relationships between cognition and internetwork coupling topologies in PD and healthy control (HC) groups. Scatterplots display the best-fitting linear regression line (solid line) and 95% conference intervals (dotted lines) for significant correlations between age adjusted cognitive measures (*y*-axis) and internetwork connectivity topologies (*x*-axis). Top four rows show relationships with baseline cognitive functioning. Bottom two rows show relationships with 2-year changes in cognition, calculated using age-adjusted simple discrepancy scores designated by Δ (score at time 1—score at time 2). Positive discrepancy scores signify cognitive decline at the follow-up visit, except for the CWINH for which negative discrepancy scores signify cognitive decline. *Predicted values from the regression of internetwork connectivity onto MoCA scores are plotted for pDMN-LFPN and pDMN-RFPN couplings [Σ intercept + (beta_pDMN–LFPN_ * pDMN-LFPN score) + (beta_pDMN–RFPN_ * pDMN-RFPN score) = Σ–0.33 + (0.08 * pDMN-LFPN score) + (0.10 * pDMN-RFPN score)]. For DOT scores, predicted values are plotted for aDMN-pDMN and aDMN-LFPN couplings [Σ intercept + (beta_aDMN–pDMN_ * aDMN-pDMN score) + (beta_aDMN–LFPN_ * aDMN-LFPN score) = Σ–0.34 + (0.09 * aDMN-pDMN score) + (0.06 * aDMN-LFPN score)]. BNT, Boston Naming Test; BVMT, Brief Visuospatial Memory Test; CVLT, California Verbal Learning Test; CWINH, Color-Word Inhibition; CWN, Color Word Naming; DOT, Adaptive Digit Ordering; HVOT, Hooper Visual Organization Test; JLOT, Judgment of Line Orientation; MoCA, Montreal Cognitive Assessment; SWA, Category Switching.

**FIGURE 3:**
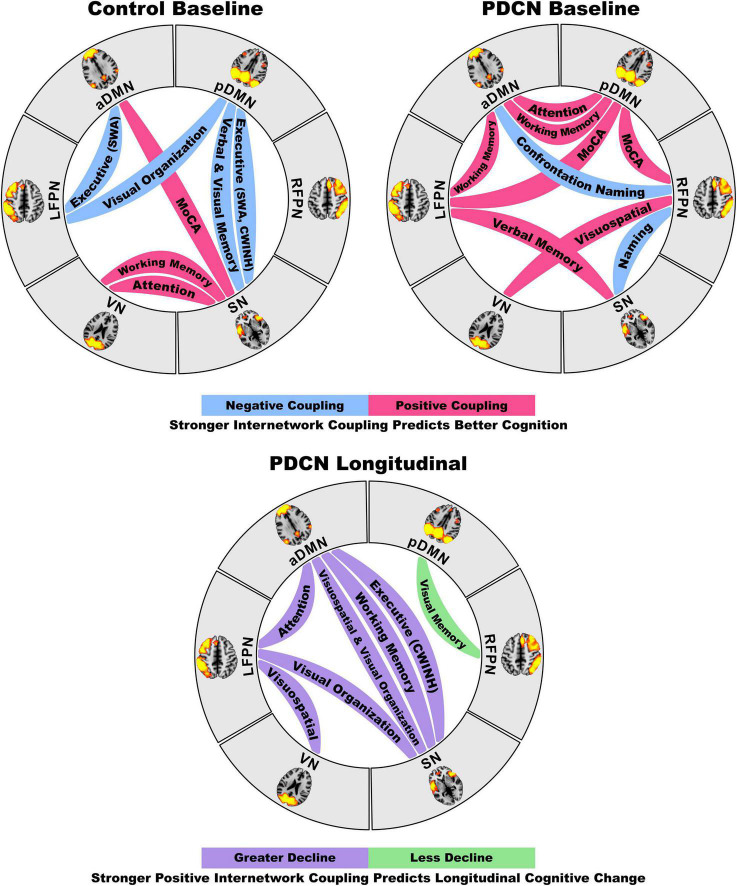
Circular visualization of internetwork functional coupling topologies as they predict baseline and longitudinal declines in cognition. The top circular layouts depict the correlations between internetwork coupling topologies and baseline domain-specific cognitive performance in the healthy control and PDCN groups. Blue lines designate stronger negative (anticorrelated) couplings and red lines designate stronger positive couplings were correlated with better cognition. The bottom circular layout shows that stronger positive internetwork couplings predict greater longitudinal decline (purple lines) for most cognitive domains, but less longitudinal decline in visual memory. These neurocognitive relationships are graphed in Figure 2.

Figure 2 displays the results from the stepwise multiple regressions, which tested for sets of internetwork couplings that best predicted baseline cognitive changes in each group separately (top) and longitudinal cognitive changes in the PDCN subsample (bottom). Graphs are shown only for results that survived FDR correction. Figure 3 (top) provides a circular visualization of the baseline results.

#### Global Cognition

In PDCN stronger positive pDMN-LFPN (*r* = 0.36) and pDMN-RFPN couplings (r_xy.z_ = 0.28) predicted better MoCA scores [*F*(2, 58) = 7.2, *p* < 0.002, *q* = 0.006, *R* = 0.45] (one outlier omitted). In controls stronger positive aDMN-SN couplings predicted better MoCA scores [*F*(1, 41) = 6.5, *p* < 0.015, *q* = 0.02, *R* = 0.37].

#### Attention and Working Memory

In PDCN stronger positive aDMN-pDMN couplings predicted better attention (CWN) [*F*(1, 61) = 4.9, *p* < 0.03, *q* = 0.045, *R* = 0.27], whereas in controls stronger positive VN-SN couplings predicted better attention [*F*(1, 41) = 5.6, *p* < 0.02, *q* = 0.03, *R* = 0.35]. In PDCN stronger positive aDMN-pDMN (*r* = 0.28) and aDMN-LFPN (r_xy.z_ = 0.25) couplings predicted better working memory (DOT) [*F*(2, 60) = 4.7, *p* < 0.01, *q* = 0.019, *R* = 0.37], whereas in controls stronger positive VN-SN couplings predicted better working memory [*F*(1, 41) = 10.0, *p* < 0.003, *q* = 0.008, *R* = 0.44].

#### Executive

Internetwork couplings in PDCN did not predict executive functioning. In controls stronger positive pDMN-SN [*F*(1, 41) = 5.9, *p* < 0.02, *q* = 0.03, *R* = 0.35] and aDMN-LFPN [*F*(1, 41) = 8.7, *p* < 0.005, *q* = 0.01, *R* = 0.42] couplings correlated with poorer category switching accuracy (VFSWA). Stronger positive pDMN-SN couplings also correlated with poorer inhibitory control (CWINH) [*F*(1, 41) = 5.2, *p* < 0.029, *q* = 0.04, *R* = 0.33].

#### Episodic Memory

In PDCN stronger positive LFPN-SN couplings predicted better verbal memory (CVLT) [*F*(1, 61) = 11.3, *p* < 0.001, *q* = 0.002, *R* = 0.40]. In controls stronger positive pDMN-SN couplings predicted poorer verbal [*F*(1, 41) = 7.4, *p* < 0.01, *q* = 0.02, *R* = 0.39] and visual memory (BVMT) [*F*(1, 41) = 5.3, *p* < 0.026, *q* = 0.039, *R* = 0.34].

#### Visual Cognition

In PDCN stronger positive VN-RFPN couplings predicted better visuospatial processing (JLOT) [*F*(1, 61) = 11.4, *p* < 0.001, *q* = 0.004, *R* = 0.40]. In controls stronger positive pDMN-LFPN couplings predicted poorer visual organization (HVOT) [*F*(1, 41) = 5.0, *p* < 0.03, *q* = 0.047, *R* = 0.33].

#### Semantic Language

In PDCN stronger positive aDMN-RFPN [*F*(1, 61) = 7.5, *p* < 0.008, *q* = 0.01, *R* = 0.33] and RFPN-SN couplings [*F*(1, 61) = 5.4, *p* < 0.024, *q* = 0.037, *R* = 0.29] predicted poorer object naming (BNT). Internetwork connectivity did not predict semantic cognition in controls.

### Internetwork Connectivity Predictors of Longitudinal Changes in Cognition

Supplementary Table 2 details the demographic and clinical characteristics of 40 PDCN participants who completed longitudinal neuropsychological testing. At the follow-up testing, no patient had a clinical diagnosis of dementia. Only eight patients (20%) exhibited MCI (> –1.5 SD on two or more tests), all of whom showed multidomain cognitive impairment. The number of months between baseline and follow-up testing did not correlate with longitudinal changes of any cognitive variable (*p* > 0.20 to *p* < 0.96). Paired *t*-tests (bootstrapped) showed significantly poorer follow-up performance on the SWA, CWINH, HVOT, and CAT (Table 3). Subthreshold trends for longitudinal decline were observed for CWN, JLOT, and DOT.

**TABLE 3:** Longitudinal changes in cognition in a PDCN subsample.

	Visit 1	Visit 2	*p*	d±
Montreal Cognitive Assessment (MoCA)	27.0 (2.5)	26.7 (3.0)	0.43	0.13
**Attention and working memory**				
Adaptive Digit Ordering (DOT)	6.7 (1.9)	6.2 (2.7)	0.12	0.26
DKEFS Color + Word Naming (CWN)	21.9 (4.3)	20.9 (4.4)	0.08	0.29
**Executive (DKEFS)**				
Category switching (accuracy) (SWA)	13.6 (2.9)	11.5 (5.0)	**0.009**	0.49
Color-Word Inhibition (CWINH)	59.2 (14.2)	64.1 (22.5)	**0.04**	0.39
**Memory (long delay free recall)**				
California Verbal Learning Test 2 (CVLT)	9.5 (3.3)	8.8 (5.2)	0.26	0.18
Brief Visuospatial Memory Test-Revised (BVMT)	8.7 (2.5)	8.6 (3.0)	0.84	0.03
**Visual cognition**				
Judgment of Line Orientation (JLOT)	25.4 (2.6)	24.3 (4.5)	0.08	0.31
Hooper Visual Organization HVOT)	25.7 (2.5)	24.2 (3.6)	**0.006**	0.52
**Semantic language**				
Boston naming (BNT)	57.8 (2.6)	57.9 (3.0)	0.86	0.03
DKEFS Category Fluency (CAT)	43.9 (8.6)	38.8 (9.2)	**0.001**	0.60

*Tabled values are raw score means (standard deviations) from a subsample of 40 PDCN participants. Longitudinal changes between baseline (Visit 1) and follow-up testing (Visit 2) were analyzed using paired t-tests with bias corrected accelerated bootstrapping (1,000 iterations). Follow-up testing occurred a mean of 24.5 months (SD = 3.4) post-baseline testing: Significant p values in bold.*

*DKEFS, Delis Kaplan Executive Function System.*

*±Cohen’s d.*

Stepwise multiple regressions tested for sets of internetwork couplings that best predicted longitudinal changes in cognition as measured by age adjusted simple discrepancy scores (score at time 1—score at time 2). Positive discrepancy scores signify cognitive decline at the follow-up visit, except for the CWINH wherein negative discrepancy scores signify cognitive decline at the follow-up visit. Figure 2 (bottom) shows graphs of significant results and Figure 3 (bottom) displays a circular visualization of the findings.

#### Attention and Working Memory

Stronger positive aDMN-LFPN couplings predicted greater decline in attention (CWN) [*F*(1, 38) = 6.8, *p* < 0.01, *q* = 0.017, *R* = 0.39]. Stronger positive aDMN-SN couplings predicted greater decline in working memory (DOT) [*F*(1, 38) = 5.8, *p* < 0.02, *q* = 0.027, *R* = 0.36].

#### Executive

Stronger positive aDMN-SN couplings predicted greater decline in inhibitory control (CWINH) [*F*(1, 38) = 5.3, *p* < 0.027, *q* = 0.04, *R* = 0.35].

#### Episodic Memory

Stronger anticorrelated pDMN-RFPN couplings predicted greater decline in visual episodic memory (BVMT) [*F*(1, 38) = 4.2, *p* < 0.047, *q* = 0.05, *R* = 0.32].

#### Visual Cognition

Stronger positive VN-LFPN [*F*(1, 38) = 6.0, *p* < 0.019, *q* = 025, *R* = 0.37] and aDMN-SN couplings [*F*(1, 38) = 5.7, *p* < 0.023, *q* = 0.035, *R* = 0.36] predicted greater decline in visuospatial processing (JLOT). Stronger positive aDMN-SN [*F*(1, 38) = 5.9, *p* < 0.02, *q* = 0.03, *R* = 0.37] and LFPN-SN couplings [*F*(1, 38) = 7.5, *p* < 0.01, *q* = 0.015, *R* = 0.41] predicted greater decline in visual-organization (HVOT).

### Genetic Associations With Internetwork Connectivity and Cognitive Decline

**FIGURE 4:**
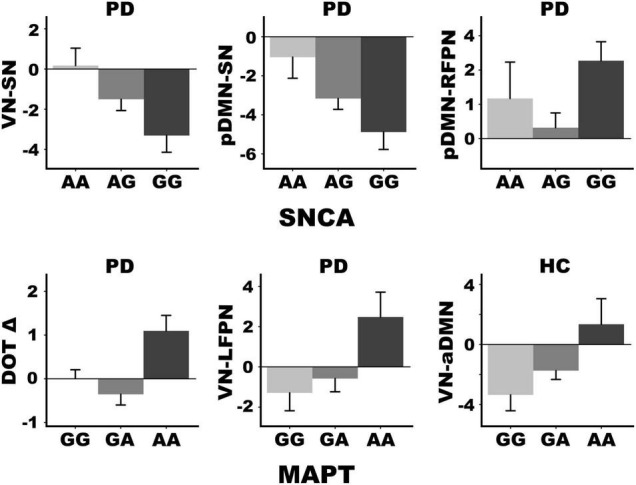
SNCA and MAPTrs242557 mediation of internetwork coupling topologies and longitudinal change in working memory. SNCA expression altered connectivity strength in the PDCN group only but was not associated with baseline or longitudinal changes in cognition. MAPT variants altered connectivity strength of different internetwork topologies in the PDCN and healthy control (HC) groups. Moreover, only PDCN A homozygotes exhibited longitudinal decline in working memory (DOT Δ) as measured by age adjusted simple discrepancy scores (score at time 1—score at time 2) wherein positive scores signify cognitive decline. Means and standard error bars are plotted.

Stepwise multiple regression analyses tested for sets of internetwork couplings that predicted SNCA and MAPT variants, separately for each group. Figure 4 graphs the significant results (FDR corrected). SNCA was not related to internetwork connectivity in healthy controls. In PDCN, stronger anticorrelated VN-SN couplings were observed in homozygous G carriers (high α-synuclein activity) than A carriers [*F*(1, 61) = 8.4, *p* < 0.005, *q* = 0.04, *R* = 0.35]. SNCA expression also was predicted by pDMN-SN (*r* = –0.36) and pDMN-RFPN (r_xy.z_ = 0.30) couplings [*F*(2, 60) = 7.9, *p* < 0.001, *q* = 0.01, *R* = 0.46]. Here, pDMN-SN anticorrelations were stronger in homozygous G than A carriers, whereas stronger positive pDMN-RFPN couplings were observed in homozygous G relative to A carriers. MAPT was related to internetwork connectivity in both groups. In PDCN stronger positive VN-LFPN couplings were observed in homozygote A carriers (high tau activity) than G carriers [*F*(1, 61) = 4.2, *p* < 0.004, *q* = 0.02, *R* = 0.26]. In controls stronger anticorrelated VN-aDMN couplings were found in G homozygotes (lower tau activity) than A carriers [*F*(1, 41) = 8.6, *p* < 0.005, *q* = 0.03, *R* = 0.42].

ANOVAs tested for genetic associations with (1) age adjusted cognitive measures at baseline in each group and (2) longitudinal changes (age adjusted simple discrepancy scores) in the PDCN subsample. Carriers of different SNCA or MAPT allele types did not differ demographically (age, educational level, gender) in either group or in clinical characteristics (disease duration, levodopa dosage equivalence). SNCA and MAPT variants were not associated with baseline cognitive measures in either group or longitudinal cognitive changes in the PDCN subsample, with one exception. Figure 4 shows that MAPT homozygous A carriers exhibited working memory (DOT) decline at follow-up testing, whereas working memory was preserved in G carriers [*F*(2, 37) = 4.9, *p* < 0.01, *q* = 0.05, η*_*p*_*^2^ = 0.21].

## Discussion

Internetwork connectivity was largely preserved in PDCN, except for reduced pDMN-RFPN/LFPN couplings, which correlated with poorer baseline global cognition. This finding may reflect a weakened role of the FPN in regulating flexible interactions with the DMN to integrate processing resources (Spreng et al., 2010) needed to achieve diverse demands of global cognition tests. Preserved internetwork interactions in PDCN also correlated with cognition, but in a different manner for each group. In PDCN, stronger positive internetwork coupling topologies were beneficial for most, but not all, cognitive functions at baseline. This dominant neurocognitive pattern was compatible with a compensatory mechanism that may arise owing to less effective integration between other networks that normally support cognition. Indeed, different internetwork coupling topologies correlated with cognition in healthy controls. Notably, stronger positive internetwork coupling topologies also predicted greater 2-year longitudinal declines in most cognitive domains in PDCN, suggesting they may signify neurodegeneration and portend future neurocognitive progression (Cabeza et al., 2018). Heterogeneity in internetwork connectivity strength was also related to genetic variants. Higher α-synuclein was associated with reduced SN-VN/pDMN interactions and amplified RFPN-pDMN couplings, which longitudinally preserved visual memory in PDCN. In contrast, higher tau expression accelerated longitudinal working memory decline and increased VN-LFPN connectivity, which in turn predicted greater decline in visuospatial processing, a risk factor for PD dementia (Williams-Gray et al., 2013). These results could not be attributed to brain atrophy, which did not differ between groups, nor aberrant LFPN intra-network coherence, which did not correlate with internetwork connectivity.

### Distinct Internetwork Topologies Predict Baseline Cognition in Cognitively Normal PD and Controls

Cognition is supported through integration and segregation (anticorrelated) of processing resources from large-scale networks (Spreng et al., 2010), which are regulated by the FPN and SN (Sridharan et al., 2008; Grady et al., 2016). In our study both groups showed positive pDMN-LFPN/RFPN couplings, albeit significantly reduced in PDCN. However, stronger pDMN-FPN couplings were beneficial for MoCA performance only in PDCN, in agreement with favorable effects of cooperative DMN-FPN communications on diverse cognitive processes (Fornito et al., 2012; Utevsky et al., 2014; Marek and Dosenbach, 2018). Cooperative FPN communications with the pDMN, which is associated with episodic memory and interrelated associative or constructive processes (Hassabis and Maguire, 2007; Andrews-Hanna et al., 2014a), may support MoCA performances especially on tests related to posterior cortical functions (e.g., visuoconstruction, memory encoding and recollection). In controls, however, positive aDMN-SN interactions benefitted MoCA performance. The aDMN consists of prefrontal and anterior temporal cortices that regulate executive, and conceptual/semantic processes (Wood and Grafman, 2003; Andrews-Hanna et al., 2014b), whereas the SN governs attention capture, internetwork switching, and inhibition. Thus, aDMN-SN communications are well suited to foster multidomain cognitive control (Sridharan et al., 2008). This coupling topology was not deployed in PDCN, possibly owing to emerging SN pathology (Christopher et al., 2015). Thus, global cognition in PDCN appears to be maintained via functional reconfiguration of internetwork communications, driven by pathological changes that cause shifts in processing load to other networks.

This conclusion aligns with our finding that internetwork connectivity correlated with domain-specific cognition differently in each group. We discovered that stronger aDMN-pDMN and aDMN-pDMN/LFPN couplings in PDCN were, respectively, favorable for attention (CWN) and working memory (DOT). The CWN test is a speeded test that requires selective attention to colors or words, whereas the DOT engages executive functions, as digit strings of increasing length must be mentally reorganized into their ascending order for recall. Both tests may benefit from stronger aDMN-pDMN connectivity owing to overlapping endogenous control and mnemonic processes, whereas favorable influences of strengthened aDMN-LFPN connectivity on the DOT aligns with LFPN control of executive components of working memory. By comparison, in healthy controls better attention and working memory both correlated with stronger VN-SN connectivity. The SN is known to drive switching between the DMN and FPN (Goulden et al., 2014). By inference, our finding may suggest that healthy adults deploy the SN to enlist VN resources to support visual attention and memory. VN-SN communications may not be effectively deployed in PDCN, perhaps owing to declining visual cognition (Weil et al., 2016), which we observed in our study, and/or emerging pathology that renders the anterior insula and anterior cingulate cortex vulnerable (Christopher et al., 2014).

Internetwork connectivity was not related to executive functioning in the PDCN group. However, stronger positive LFPN-SN and VN-RFPN couplings were compensatory, respectively, predicting better baseline verbal memory and visuospatial cognition, which exhibited decline in PDCN. In controls, however, stronger anticorrelated pDMN-SN couplings predicted better executive functioning (category switching and inhibition) and verbal/visual memory, consistent with another study (Putcha et al., 2016). Stronger anticorrelated aDMN-LFPN couplings also predicted better category switching, whereas stronger anticorrelated pDMN-LFPN couplings correlated with better visual organization. Thus, in controls segregation of task-negative and positive networks was favorable for many cognitive functions, ostensibly due to reduced competition between processes that can interfere with performance (Kelly et al., 2008).

While strengthened positive internetwork coupling topologies in PDCN typically exerted favorable influences on cognition, an exception was the BNT (confrontation naming), a measure of semantic recollection that requires generating names of common and uncommon pictures. Semantic recollection is highly relevant to PD since early decline in object naming predicts later dementia (Williams-Gray et al., 2013). We found that stronger positive aDMN-RFPN and RFPN-SN couplings in PDCN were detrimental for naming. Outwardly this finding was unexpected as integration of executive/semantic (aDMN) and attention (SN) resources with RFPN visual processing resources might foster object naming. For the BNT, however, common object names are automatically retrieved whereas uncommon object names require attentional control (Hoffman et al., 2015). One speculation is that communications of the RFPN with attention and executive/semantic networks may be amplified in patients who have difficulty finding names due to failed automatic retrieval of semantic details, which could be a sign of impoverished representations of semantic content. This proposal is compatible with a task-activated fMRI study of semantic recollection (Harrington et al., 2021). We found that stronger connectivity of a semantic hub and the caudate with regions comprising the dorsal attention, executive, and/or language systems correlated with poorer BNT performance in PDCN. These results suggested the possibility that patients with poorer naming may allocate more attention and executive resources to support covert verbal strategic searches needed to find names that cannot be retrieved automatically (Harrington et al., 2021).

### Internetwork Topologies Predict Future Cognitive Decline in Cognitively Normal PD

At 2 years post-baseline, the PDCN subsample showed significant decline in executive functioning, visual organization, and semantic fluency, with subthreshold trends for decline in attention, working memory and visuospatial processing. Greater cognitive decline for most domains, except visual episodic memory, was predicted by strengthened positive internetwork couplings. Thus, strengthened internetwork couplings, even those exerting compensatory influences on baseline cognition, foreshadowed future cognitive progression, indicating they signify neuropathology. This proposal aligns with the positive correlation between increased cortical connectivity and glucose consumption (FDG-PET) (Tomasi et al., 2013; Passow et al., 2015), which indicates a metabolic cost for hyperconnectivity that may in turn signify neuronal vulnerability. Indeed, cognitively normal people at genetic risk for Alzheimer’s initially exhibit hyperactivation in the brain relative to people not at genetic risk, but activation declines longitudinally together with the emergence of episodic memory dysfunction (Rao et al., 2015).

Some internetwork topologies predicted different facets of cognition at baseline and longitudinally, likely because general processing resources are shared across domains, some of which are more vulnerable to cognitive progression (Salthouse, 2017; Tucker-Drob et al., 2019). For example, stronger positive aDMN-LFPN and LFPN-SN, respectively, predicted greater deterioration in attention and visual organization, but better baseline working memory and visual memory. In most instances, predictors of longitudinal cognitive decline did not correlate with baseline cognition. Notably, greater decline in attention, working memory, executive, visuospatial, and visual organization functions were predicted by stronger positive aDMN-SN/LFPN and LFPN-SN couplings. Amplified internetwork couplings of these networks likely foreshadow longitudinal cognitive decline across multiple domains, owing to frontostriatal vulnerabilities driven by dopaminergic loss and SN pathology (Christopher et al., 2014). Our results contrast with a seed-based approach in which increased SN-DMN regional couplings in a PD cohort unscreened for MCI predicted conversion to dementia 10 years later (Aracil-Bolanos et al., 2021). While DMN-SN coupling strength did not correlate with PDCN cognition in our study, increased pDMN-SN connectivity was maladaptive for executive functioning and episodic memory in healthy controls, consistent with these results.

Stronger positive VN-LFPN couplings also predicted greater decline in visuospatial processing. Interestingly, VN-FPN connectivity increased over 1 year in a mixed PDCN/PDMCI cohort, but not healthy controls (Klobusiakova et al., 2019), and did not correlate with cognition. Our finding indicates this internetwork coupling topology is an early signature of high-level visual disturbances in PD (Weil et al., 2016), which is of keen interest as early visuospatial decline is a risk for MCI and dementia (Williams-Gray et al., 2013; Hong et al., 2014; Hobson and Meara, 2015; Garcia-Diaz et al., 2018; Chung et al., 2020; Ohdake et al., 2020).

The above results sharply contrasted with our finding that stronger positive pDMN-RFPN couplings predicted preserved visual episodic memory longitudinally, even though performance was poorer in PDCN relative to controls at baseline. Thus, integration of pDMN memory resources with RFPN visual processing resources appears to maintain visual memory over 2 years. Interestingly, in a PDCN/PDMCI cohort the strength of DMN-FPN couplings increased over 1 year but did not correlate with cognition (Klobusiakova et al., 2019). The latter finding may be due to the short follow-up period, which could minimize changes. However, our results also demonstrate the importance of distinguishing between left and right FPN internetwork couplings as they correlated differently with cognitive tests that emphasize verbal and visual processes.

### α-Synuclein and Microtubule-Associated Protein Tau Alter Cognition and Internetwork Connectivity

The SNCA rs356219 polymorphism codes for α-synuclein levels, with the G risk allele associated with higher transformed plasma α-synuclein (Mata et al., 2010; Emelyanov et al., 2018), reduced striatal binding potential (Huertas et al., 2017), and increased risk for PD (Goris et al., 2007; Trotta et al., 2012; Zhang et al., 2018). Although SNCA does not predict dementia outcome over 10 years (Williams-Gray et al., 2013), its association with cognitive decline is controversial, especially in PDCN. In our study, SNCA did not mediate baseline or longitudinal changes in cognition, consistent with some studies (Mata et al., 2014; Huertas et al., 2017), but not others (Campelo et al., 2017; Luo et al., 2019). Yet another SNCA risk variant (rs894280) predicted poorer attention and visuospatial processing (Ramezani et al., 2021). Correspondingly, higher baseline CSF α-synuclein levels also predicted faster longitudinal deterioration in visuospatial working memory and verbal memory (Stewart et al., 2014) and processing speed (Hall et al., 2015). Our discrepant results may be related to studying a PDCN cohort wherein cognition is intact relative to cohorts not screened for MCI.

Nonetheless, we found for the first time that SNCA mediated the strength of functional connectivity between large-scale networks, but only in PDCN. This finding is compatible with the SNCA risk variant’s role in modifying the effect of CSF α-synuclein levels on brain structure by accelerating cortical thinning throughout frontal and posterior cortical areas (Sampedro et al., 2018a). In our study, homozygous G carriers showed marked anticorrelated SN-VN and SN-pDMN couplings relative to A carriers. As these topologies were associated with cognition only in healthy controls, our results suggest that the SNCA risk variant amplifies segregation between the SN and the VN/pDMN, rendering their deployment less effective for cognition. Thus, accumulation of α-synuclein pathology (Christopher et al., 2015) may diminish flexible SN internetwork switching (Goulden et al., 2014), thereby dampening internetwork communications. Interestingly, homozygous G carriers also exhibited stronger positive pDMN-RFPN couplings relative to A carriers. This internetwork topology was favorable for baseline MoCA scores and predicted preserved visual episodic memory longitudinally. Still, more tightly coupled compensatory pDMN-RFPN communications in GG carriers may occur at a metabolic cost (Tomasi et al., 2013; Passow et al., 2015), such that over longer periods of time compensation might diminish as neurodegeneration increases and cognitive deficits emerge (Reuter-Lorenz and Park, 2014). This prospect aligns with a report of 2-year increases in CSF α-synuclein levels only in PD patients with longer disease durations (> 5 years) (Hall et al., 2016). Thus, longer follow-up periods may be needed to better characterize the trajectory of neurocognitive progression in carriers of risk and protective SNCA alleles.

MAPT had independent effects on internetwork communications and cognitive decline. The MAPT H1 haplotype promotes tau aggregation, which may interact with α-synuclein in Lewy body formation (Colom-Cadena et al., 2013). Higher tau transcription in PD appears to accelerate cognitive decline in early years of PD and is the strongest genetic marker of dementia conversion (Williams-Gray et al., 2013). In our study, H1 tau transcription levels had no effect on baseline cognition, consistent with some studies of the MAPT H1/H2 diplotype (Mata et al., 2014; Paul et al., 2016) but not others (Morley et al., 2012; Nombela et al., 2014). For the first time, we found working memory decline was strongly mediated by the A risk allele, which is associated with higher plasma tau levels (Chen et al., 2017), whereas working memory was preserved in G carriers. Correspondingly, tau deposition in older adults is found in large-scale brain networks including the FPN (Jones et al., 2017), which supports working memory. We also found group differences in MAPT-mediated internetwork coupling topologies. In PDCN, homozygous A carriers exhibited stronger positive VN-LFPN couplings whereas G carriers showed negative couplings. Importantly, stronger positive VN-LFPN couplings predicted greater longitudinal decline in visuospatial processing, another risk factor for the later MCI and dementia. These findings partly agree with the effect of the MAPT H1 haplotype on longitudinal volume loss in *de novo* PD, largely in the frontal cortex (Sampedro et al., 2018b), which is an element of the LFPN. The results are also compatible with reports that MAPT variants alter activation and functional connectivity of the parietal cortex, which is an element of both the VN and LFPN. Specifically, PD carriers of the MAPT H1 haplotype showed reduced left parietal and bilateral caudate activation relative to non-risk carriers when performing a mental rotation task (Nombela et al., 2014). Correspondingly, during a semantic recollection task PDCN carriers of the MAPT A risk allele showed stronger left parietal connectivity with the bilateral caudate, which was unfavorable for cognition, but diminished frontal connectivity with parietal areas, which supported compensation (Harrington et al., 2021). Altogether, our finding aligns with the vulnerability of occipital and frontoparietal cortices to tau deposition (Robakis et al., 2016; Jones et al., 2017), and implicates increased tau activity in the acceleration of visuospatial decline. In contrast, healthy control homozygote A carriers showed stronger positive VN-aDMN couplings whereas G carriers showed negative couplings, suggesting that tau deposition in occipital cortex may also drive aberrant VN internetwork communications in older adults.

### Limitations

Limitations include that testing patients on medication therapy could mask functional abnormalities and affect performance on neuropsychological tests. Head motion artifact, however, can be elevated off medication, which has detrimental influences especially on low frequency rsfMRI signals. Completion of a lengthy neuropsychological test battery can also be challenging for patients after overnight withdrawal from medication therapy. From a practical standpoint it is also important to understand brain functioning and cognition in daily life as influenced by medication therapy. Second, the inclusion of six *de novo* PDCN patients increased the heterogeneity within the cohort, which could add variability to the functional connectivity measures. Despite this limitation, internetwork coupling topologies remained sensitive to cognitive and genetic variables, likely owing in part to the improved temporal resolution of our multiband fMRI protocol (Tomasi et al., 2016). Third, neurocognitive correlations were medium in magnitude, likely owing to the more restricted ranges on behavioral variables in a PDCN cohort, which may partly relate to the use of compensatory strategies that maintain cognition (Reuter-Lorenz and Park, 2014). Fourth, although our sample size was large, the statistical power for tests of genetic variants would be improved with larger samples. Even so, medium effect sizes were found for MAPT and SNCA effects on internetwork connectivity and a very large effect size was observed for MAPT prediction of working memory decline. Fifth, although our longitudinal analyses controlled for aging effects, it would be desirable to collect the same neuropsychological data longitudinally in elder controls to better gauge the rate of disease-related cognitive progression. Lastly, our analyses were constrained to six core large-scale networks, but other networks should also be considered to fully characterize internetwork communications that predict domain-specific cognitive progression.

## Conclusion

Our results show that cognition in PDCN is maintained by functional reconfiguration of internetwork communications, possibly driven by underlying pathology that causes a shift in processing resources to other networks. We demonstrated for the first time that stronger positive internetwork coupling topologies exerted mainly compensatory influences on baseline cognition in PDCN, but predicted longitudinal changes in most cognitive domains, suggesting that they are surrogate markers of neuronal vulnerability. Strengthened aDMN-SN, LFPN-SN, and/or LFPN-VN interactions predicted longitudinal decline in visual cognition, a risk factor for future MCI and dementia, and declines in attention, executive functioning, and working memory, which are processes shared across multiple cognitive domains. Coupling strengths of some internetwork topologies were altered by genetic variants. The main findings showed that higher α-synuclein weakened internetwork communications of the SN that supported cognition in healthy controls, whereas higher tau increased VN-LFPN connectivity, which in turn predicted greater longitudinal visuospatial decline, a risk factor for dementia. Notably, the tau risk variant also accelerated longitudinal decline in working memory, whereas the protective allele prevented working memory decline. Still, internetwork coupling topologies did not predict longitudinal change in semantic fluency, which was robust in the PDCN group and is also a risk factor for MCI and dementia (Compta et al., 2013; Williams-Gray et al., 2013; Hobson and Meara, 2015). This is likely because our ICA analyses did not expose a temporal-parietal network, for which key hubs (angular gyrus, temporal pole) modulate semantic fluency in PDCN (Harrington et al., 2021). Collectively, these novel findings emphasize the prognostic value of large-scale internetwork connectivity in predicting domain-specific cognitive decline and the distinct modulatory influences of SNCA and MAPT, which partly explain heterogeneity in neurocognition. Future investigations into the roles of other genetic variants in neurocognitive functioning would be of great interest, given the paucity of studies concerning genetic modifiers of functional connectivity in PDCN.

## Data Availability Statement

The data that support the findings of this study are available on reasonable request from the corresponding author. The data are not publicly available due to privacy or ethical restrictions imposed the U.S. Department of Veteran Affairs. Genetic variant data from this study are deposited in dbSNP (https://www.ncbi.nlm.nih.gov/SNP/snp_viewTable.cgi?handle=COGNITIONPD).

## Ethics Statement

The studies involving human participants were reviewed and approved by the Institutional Review Board at the VA San Diego Healthcare System. The patients/participants provided their written informed consent to participate in this study.

## Author Contributions

XW wrote the first draft of the manuscript and performed the neuroimaging analyses. QS supervised and assisted with the neuroimaging analyses and acquired MRI data. RL reviewed brain MRIs. XW, IL, QS, MH, and RL contributed critical content to the manuscript revision. DH conceived and designed the study, performed statistical analyses, and wrote the final manuscript. All authors read and approved the submitted version.

## Conflict of Interest

The authors declare that the research was conducted in the absence of any commercial or financial relationships that could be construed as a potential conflict of interest.

## Publisher’s Note

All claims expressed in this article are solely those of the authors and do not necessarily represent those of their affiliated organizations, or those of the publisher, the editors and the reviewers. Any product that may be evaluated in this article, or claim that may be made by its manufacturer, is not guaranteed or endorsed by the publisher.

## Footnotes

1
https://www.dnagenotek.com

2
http://www.fmrib.ox.ac.uk/fsl/

3
https://afni.nimh.nih.gov/

4
http://surfer.nmr.mgh.harvard.edu
