# Supplementary material for: Internetwork Connectivity Predicts Cognitive Decline in Parkinson’s and Is Altered by Genetic Variants
Source: Front Aging Neurosci. 2022 Mar 28;14:853029. doi: 10.3389/fnagi.2022.853029 (PMC8996114; doi:10.3389/fnagi.2022.853029)
Supplement: Supplementary file 1 [file Table_1.DOCX]

**Supplementary Table 1. Main regions of independent component networks.**

|  | **MNI Coordinate** | | |  |
| --- | --- | --- | --- | --- |
| **Independent Component Networks** | **X** | **Y** | **Z** | **Z score** |
| **Visual Network (VN)** |  |  |  |  |
| L Superior Occipital | -21 | -83 | 29 | 7.27 |
| R Superior Occipital | 21 | -83 | 29 | 6.87 |
| L Middle Occipital | -28 | -78 | 26 | 6.61 |
| R Middle Occipital | 28 | -78 | 26 | 6.95 |
| L Cuneus | -4 | -82 | 21 | 8.60 |
| R Cuneus | 4 | -82 | 21 | 7.96 |
| L Precuneus | -8 | -76 | 21 | 8.36 |
| R Precuneus | 8 | -76 | 21 | 7.86 |
| **anterior Default Mode Network (aDMN)** |  |  |  |  |
| Medial Prefrontal | 0 | 60 | 25 | 11.41 |
| L Superior Frontal | -19 | 60 | 20 | 11.26 |
| R Superior Frontal | 17 | 59 | 20 | 9.03 |
| L Middle Frontal | -31 | 47 | 22 | 4.78 |
| R Middle Frontal | 31 | 49 | 22 | 3.81 |
| L Precuneus | -5 | -54 | 30 | 4.94 |
| R Precuneus | 5 | -54 | 30 | 4.14 |
| L Posterior Cingulate | -6 | -56 | 16 | 4.14 |
| R Posterior Cingulate | 6 | -56 | 16 | 3.53 |
| L Angular Gyrus | -49 | -62 | 29 | 5.06 |
| R Angular Gyrus | 49 | -62 | 29 | 3.02 |
| L Temporal Pole | -41 | 24 | -28 | 6.60 |
| R Temporal Pole | 41 | 24 | -28 | 6.38 |
| **posterior Default Mode Network (pDMN)** |  |  |  |  |
| L Angular Gyrus | -45 | -64 | 33 | 12.31 |
| R Angular Gyrus | 45 | -64 | 33 | 8.83 |
| L Precuneus | -10 | -62 | 34 | 9.17 |
| R Precuneus | 10 | -62 | 34 | 9.31 |
| L Posterior Cingulate | -6 | -48 | 27 | 5.18 |
| R Posterior Cingulate | 6 | -48 | 27 | 6.64 |
| L Inferior Parietal | -45 | -64 | 43 | 10.87 |
| R Inferior Parietal | 45 | -64 | 43 | 10.69 |
| L Superior Parietal | -36 | -68 | 53 | 9.77 |
| R Superior Parietal | 36 | -68 | 53 | 8.05 |
| L Middle Temporal | -66 | -40 | -4 | 5.70 |
| R Middle Temporal | 66 | -40 | -4 | 7.50 |
| Medial Frontal | 0 | 35 | 43 | 4.22 |
| L Middle Frontal | -26 | 25 | 43 | 4.15 |
| R Middle Frontal | 26 | 25 | 43 | 4.53 |
| **Left Frontoparietal Network (LFPN)** |  |  |  |  |
| L Dorsal-lateral Prefrontal | -37 | 40 | 26 | 9.22 |
| L Middle Frontal | -42 | 27 | 26 | 7.79 |
| L Inferior Frontal | -44 | 21 | 4 | 5.33 |
| L Medial Superior Frontal | -3 | 27 | 57 | 5.12 |
| L Inferior Parietal | -55 | -37 | 39 | 9.05 |
| L Superior Parietal | -33 | -68 | 51 | 4.73 |
| L Precuneus | -32 | -68 | 41 | 4.35 |
| L Middle Temporal | -60 | -44 | -5 | 5.90 |
| **Right Frontoparietal network (RFPN)** |  |  |  |  |
| R Dorsal-lateral Prefrontal | 37 | 40 | 26 | 7.57 |
| R Middle Frontal | 45 | 30 | 26 | 6.88 |
| R Inferior Frontals | 39 | 21 | 5 | 4.95 |
| R Medial Superior Frontal | 3 | 26 | 46 | 6.26 |
| R Inferior Parietal | 55 | -39 | 47 | 6.70 |
| R Superior Parietal | 35 | -63 | 51 | 5.68 |
| R Precuneus | 32 | -65 | 41 | 4.69 |
| R Middle Temporal | 60 | -44 | -8 | 4.74 |
| **Salience Network (SN)** |  |  |  |  |
| L Anterior Insula | -46 | 10 | 1 | 5.53 |
| R Anterior Insula | 46 | 10 | 1 | 5.28 |
| Caudal Anterior Cingulate | 0 | 40 | 20 | 5.02 |
| L Frontal Operculum | -44 | 17 | 2 | 5.47 |
| R Frontal Operculum | 44 | 17 | 2 | 5.78 |
| Supplementary Motor Area | 0 | 3 | 58 | 5.65 |

Z scores reflect the magnitude of connectivity of a regional voxel within the network.
