# Supplementary material for: Internetwork Connectivity Predicts Cognitive Decline in Parkinson’s and Is Altered by Genetic Variants
Source: Front Aging Neurosci. 2022 Mar 28;14:853029. doi: 10.3389/fnagi.2022.853029 (PMC8996114; doi:10.3389/fnagi.2022.853029)
Supplement: Supplementary file 2 [file Table_2.DOCX]

**Supplementary Table 2. Characteristics of the PDCN subsample.**

| **Baseline demographic, clinical, & genetic characteristics** | | | | |
| --- | --- | --- | --- | --- |
| Age (years) | 64.7 (7.1) |  |  |  |
| Education (years) | 17.0 (2.1) |  |  |  |
| Sex (% females) | 40.00% |  |  |  |
| Disease duration (years) | 4.2 (3.4) |  |  |  |
| Months between baseline & follow up | 24.5 (3.2) |  |  |  |
| SNCA rs356219 AA:AG:GG | 7:23:10 |  |  |  |
| MAPT rs242557 GG:GA: AA | 19:16:5 |  |  |  |
| **Medication & motor severity** | **Visit 1** | **Visit 2** | **p** | **d^±^** |
| Levodopa dosage equivalence † | 799.2 (660.5) | 1139.9 (788.1) |  |  |
| UPDRS Part III | 25.3 (12.4) | 29.6 (14.6) | 0.001 | 0.63 |

Tabled values are means (standard deviations) from a subsample of 40 PDCN participants. For SNCA and MAPT variants, the frequency of each allele type is listed. Longitudinal changes between baseline (Visit 1) and follow-up (Visit 2) testing were analyzed using paired t tests with bias corrected accelerated bootstrapping (1,000 iterations).

UPDRS = Movement Disorder Society Unified Parkinson’s Disease Rating Scale

**^†^** Levodopa dosage equivalence was calculated using the method of Tomlinson (Tomlinson et al., 2010). Data are based on 36 participants who were taking dopaminergic medications at baseline and 38 participants who were taking medications at the follow-up testing period.

± Cohen’s d
